# Testing the devil’s impact on southern Baltic and North Sea basins whitefish (*Coregonus* spp.) diversity

**DOI:** 10.1186/s12862-018-1339-2

**Published:** 2018-12-29

**Authors:** Thomas Mehner, Kirsten Pohlmann, David Bittner, Jörg Freyhof

**Affiliations:** 1 Leibniz-Institute of Freshwater Ecology and Inland Fisheries, Müggelseedamm 310, 12587 Berlin, Germany; 2 Department of Fish Ecology & Evolution, Centre of Ecology, Evolution and Biogeochemistry, EAWAG Swiss Federal Institute of Aquatic Science and Technology, Seestrasse 79, 6047 Kastanienbaum, Switzerland; 3 Present address: Kanton Aargau, Departement Bau, Verkehr und Umwelt, Abteilung Wald, Jagd und Fischerei, Entfelderstrasse 22, 5001 Aarau, Switzerland

**Keywords:** *Coregonus*, Microsatellite markers, Stocking, Conservation, Admixture

## Abstract

**Background:**

The diversity and phylogeny of whitefish of the genus *Coregonus* is complex, and includes many endemic species of high conservation concern. However, because of commercial importance of whitefish fisheries, stockings and translocations have occurred repeatedly, which challenges the identification of local populations as conservation units. This study analyses the phylogenetic relationships of 15 contemporary and two historical populations of lake-resident and anadromous whitefish (*Coregonus* spp.) from the southern Baltic and North Sea basins. We elucidated the complex history of Lake Schaal (northern Germany) whitefish, for which a local tale suggests that the devil threw whitefish from the Central European Lake Constance into this lake. Studies from the early twentieth century indeed suggested numerous stocking events for Lake Schaal from Lake Constance, from Estonian/Russian Lake Peipsi and from the anadromous whitefish of the Baltic Sea.

**Results:**

Analyses of 13 microsatellite markers showed that Lake Constance whitefish are unrelated to any northern Germany whitefish population, including the contemporary whitefish population from Lake Schaal. Comparison with four historical specimens further showed that the native Lake Schaal whitefish (*C. holsatus*) vanished from the lake, but has survived as a non-native population in the north German Lake Drewitz. The whitefish currently occurring in Lake Schaal and three adjacent lakes are identified as *C. maraenoides*, introduced from Lake Peipsi. The contemporary anadromous whitefish populations from the Baltic (German and Finnish coast) and the German River Treene (North Sea basin, stocked from Danish River Vida) grouped together, but showed significant genetic differentiation. The 14 historical specimens of *C. oxyrinchus* from Rivers Rhine and Schelde were assigned to several contemporary whitefish populations, but among them only one specimen was assigned to the contemporary River Treene population. Therefore, we do not support the view that the whitefish from River Vida/Treene are identical with the historical *C. oxyrinchus*.

**Conclusions:**

Our study demonstrates that lake and anadromous whitefish in the Baltic and North Sea basins reflect a complex phylogeography, which is further blurred by the effects of repeated stocking and translocations. To identify conservation units, the genetic identity of each population has to be scrutinized.

**Electronic supplementary material:**

The online version of this article (10.1186/s12862-018-1339-2) contains supplementary material, which is available to authorized users.

## Background

Whitefish of the genus *Coregonus* represent a large group of medium to large-sized resident or migratory freshwater fishes. Their diversity and phylogeny are still largely unresolved and traditionally considered a nightmare when it comes to the recognition of species and conservation units [1–8]. Understanding the diversity of whitefish is complicated by three major problems. First, *Coregonus* species are notorious for postglacial (< 12.000 years) adaptive radiations [9, 10] and parallel evolution [11] leading to local endemic species and species flocks [12–14]. Secondly, whitefish possess only a limited number of distinguishing morphological features, certainly due to the relatively recent evolutionary divergence of many individual species and populations. The most prominent meristic character is the number of gill rakers, while other characters are more subtle and often ignored. However, the number of gill rakers may undergo rapid modifications when environmental conditions change [15], and their reliability as taxonomic character has to be assessed for each population with great care. Thirdly, due to their high commercial value, stockings from multiple origins even over large spatial distances have been reported for *Coregonus* since the fourteenth century [16–18], but details are usually poorly documented and stocking history is rarely retraceable. Accordingly, current populations may comprise a mix of native and introduced genotypes due to introgressive hybridization [19].

A typical example of a complex and unresolved genetic history of whitefish is the locally famous case of Lake Schaal in northern Germany (area 24 km^2^, maximum depth 72 m, weakly eutrophic). At about 1920, a native origin was assumed by Thienemann [20, 21], who described the whitefish from Lake Schaal and the adjacent Lake Selenter (geographical distance about 90 km) morphologically in detail and, according to the low number (23–27) of short gill rakers, named it as a species of its own, *Coregonus holsatus*. In 1997, Kottelat [22] suggested that *C. holsatus* from Lake Schaal might be conspecific with *Coregonus widegreni*, known as the valaamka whitefish, a large deepwater species from Russian Lake Ladoga, and the Scandinavian sandsik or sea-spawning whitefish [23].

However, there are numerous reports, which indicate potential stocking activities for Lake Schaal from other sources. Documentations from the 19th and early 20th centuries report the introduction of *Coregonus maraena*, an anadromous whitefish of Baltic Sea tributaries, into Lake Schaal [24, 25]. Surprisingly, the same authors also reported that Lake Schaal was stocked with whitefish from Lake Constance, especially *Coregonus wartmanni* and *C. macrophthalmus* [16, 17]. However, the *C. holsatus* whitefish from Lake Schaal originally described by Thienemann [20, 21] morphologically strongly resemble a third of the Lake Constance whitefish species, *Coregonus arenicolus*, by their subterminal mouth, a large size of more than 450 mm standard length, 23–27 short gill rakers (18–27 in *C. arenicolus*) and a predominantly benthic foraging behaviour [18]. A potential stocking history of Lake Schaal from Lake Constance is also reflected by a local tale: An abbess in the monastery of the village Zarrentin at Lake Schaal promised her soul to the devil in return for Lake Constance whitefish during fasting. Due to strong compunctions she tricked the devil who delivered the fish from Lake Constance. The devil furiously threw the whitefish into Lake Schaal, where they were caught ever since. This myth suggests that Lake Schaal whitefish *C. holsatus* is not of native origin, but has been introduced from Lake Constance already during the period of operation of the monastery on Lake Schaal (between 1246 and 1553). Subsequently, we refer to this tale as the “devil hypothesis”.

To further complicate the situation, the lake-resident *Coregonus maraenoides* from Lake Peipsi (Estonia/Russia) have been introduced into Lake Schaal during the 1930s [26, 27], after the description of *C. holsatus* from Lake Schaal by Thienemann [20, 21]. Present-day whitefish from Lake Schaal and the nearby located Lake Selenter are morphologically similar to *C. maraenoides* (35–45 gill rakers, lower jaw slightly protruding) [28], and do no longer agree with the original description of *C. holsatus* [20, 21] or the historical *C. holsatus* material deposited at the Zoological Museum of Berlin, Germany (ZMB). In contrast, a large whitefish population, which matches all morphological characters with Thienemann’s [20, 21] original description of *C. holsatus* and the corresponding material at ZMB, exists at present in the north-east German Lake Drewitz (synonymous name Alt-Schweriner See). According to unpublished reports, whitefish, presumably *C. holsatus*, were translocated from Lake Schaal into Lake Drewitz in 1931, before stocking of *C. maraenoides* into Lake Schaal commenced [26].

Finally, *Coregonus oxyrinchus*, an anadromous whitefish from the North Sea is another potential source, which may have contributed to the stocking history of Lake Schaal. Historically, *C. oxyrinchus* has occurred in Rivers Rhine and Schelde, but meanwhile there is evidence that it is globally extinct [29]. Currently, whitefish from the Danish River Vida are stocked into Rivers Rhine and Treene, the latter being geographically in close proximity to Lake Schaal. There is an ongoing discussion about the genetic allocation of the River Vida whitefish, which is denominated and stocked as *C. oxyrinchus* [30, 31], although the genetic relationship to other anadromous whitefish populations are unresolved [29].

For nature conservation, *Coregonus* populations are of special importance, because they often represent unique species flocks [9, 10, 13, 32] or locally endemic species which are vulnerable to anthropogenic impacts [12, 14, 33]. The fragility of recent radiations to human-mediated eutrophication as well as gene flow from stocking has been highlighted for whitefish from Alpine lakes [34]. Accordingly, a valid conservation status of the current Lake Schaal population can only be obtained if genetic modification by stocking or translocation can be excluded. The phylogeography of the historical Lake Schaal whitefish population (= *C. holsatus*) is still unresolved, but molecular methods may identify potential source populations to evaluate the conservation status of whitefish in this lake.

In this study, we elucidate the phylogeographic history of the Lake Schaal whitefish population in comparison with other whitefish populations by molecular markers. We test specifically whether the present Lake Schaal population is genetically most similar to species from Lake Constance (i.e. *C. wartmanni*, *C. arenicolus*, or *C. macrophthalmus*, the “devil hypothesis”), or is more similar to and thus may originate from other European whitefish populations (i.e. from *C. maraenoides*, *C. maraena*, *C. oxyrinchus* or *C. widegreni*). In contrast, a native origin would be supported by genetic similarity with the presumably translocated contemporary *C. holsatus* population in Lake Drewitz and by correspondence with historical *C. holsatus* specimens as found in ZMB. To resolve the genetic uncertainty of the River Vida whitefish population, we also included historical museum specimens of *C. oxyrinchus* from the Rivers Rhine and Schelde populations. By including this broad variety of potential origins and the mix of contemporary populations and historical individuals, our study is the first opportunity to review the diversity of whitefish populations from the south-western Baltic and eastern North Sea basins for conservation purposes.

## Methods

### *Coregonus* species in this study

**Fig. 1:**
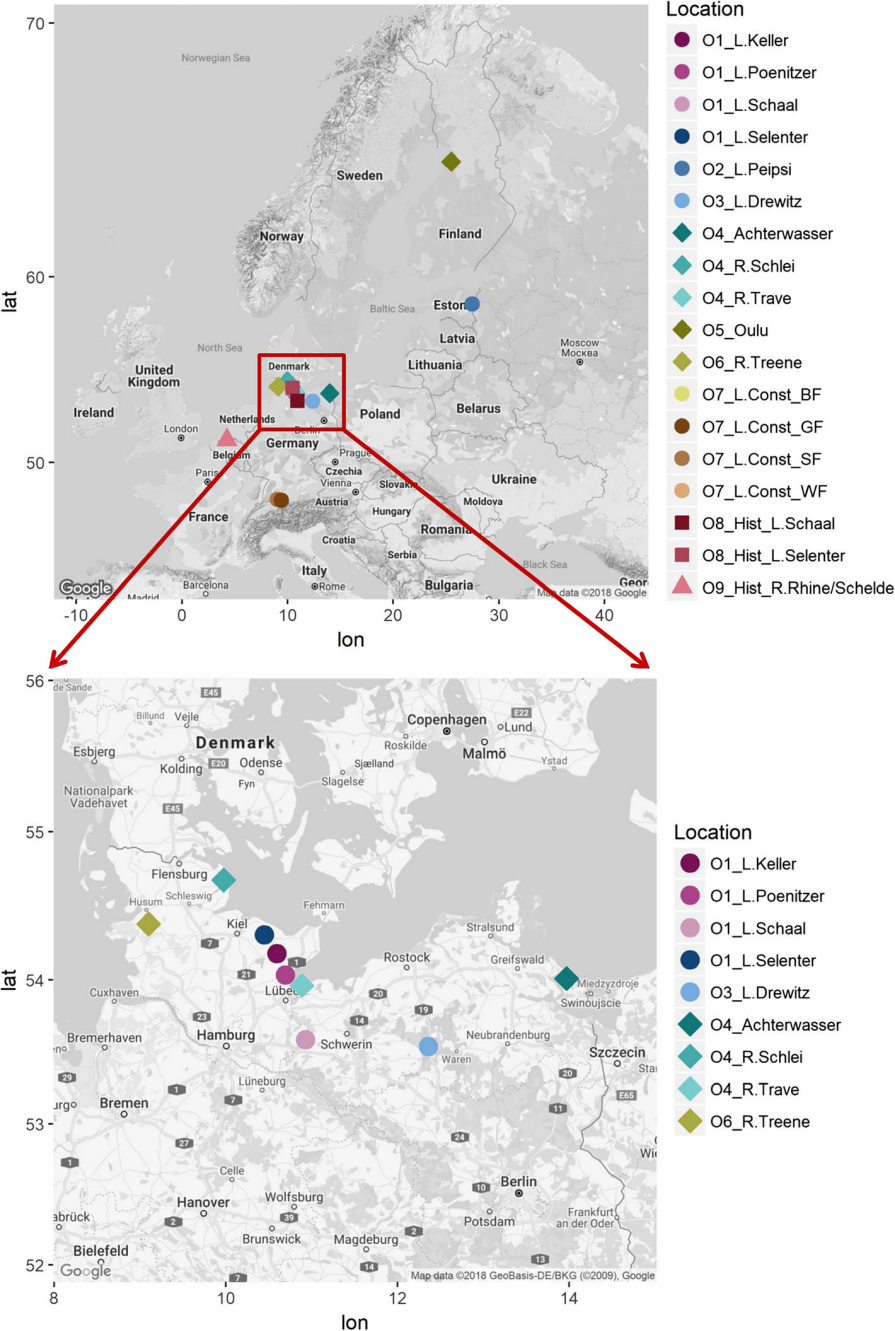
Geographical map of Europe, showing the sampling locations of 15 contemporary and 2 historical whitefish populations, accumulated into nine groups of origin (O1-O9, upper panel), and locations of population origin in northern Germany (lower panel). Lake-resident populations are indicated by circles, whereas anadromous populations are indicated by diamonds. Historical lake populations are indicated by squares and historical anadromous population by triangles. For details of populations, see Table 1

We considered the genetic relationships among nine groups of whitefish populations to elucidate the potential origin of contemporary Lake Schaal whitefish (see overview and number of specimens per population in Table 1). At about 1920, *Coregonus holsatus* was only reported from the adjacent Lakes Schaal and Selenter (geographical distance about 90 km), and therefore we obtained specimens of the contemporary populations from these two lakes. There was no distinct information about the presence of *C. holsatus* in other nearby lakes in north-west Germany, but we included samples from whitefish populations in Lakes Poenitzer and Keller, both adjacent to Lake Selenter. These four lakes form the first group of potential origin (O1, Fig. 1, Additional file 1: Table S1). We further included *C. maraenoides* from Russian/Estonian Lake Peipsi (group O2, Fig. 1), which were introduced into Lake Schaal during the 1930s [26]. Whitefish from Lake Drewitz, which probably originate from Lake Schaal [26], form the third group (O3, Fig. 1). Since the introduction of *C. maraena* into Lake Schaal is also possible, we included three *C. maraena* populations, originating from the German Baltic Sea coast (Achterwasser) and the Rivers Schlei and Trave, both draining into the Baltic Sea (group O4, Fig. 1, Additional file 1: Table S1). In 1997, Kottelat [22] suggested that *C. holsatus* and *C. widegreni* might be related and indeed, Baltic *C. widegreni* might have invaded Lakes Schaal and Selenter after the last glaciation. We thus further elucidated the phylogenetic relationship of the Lake Schaal population to *C. widegreni* obtained from the Baltic Sea coast near Oulu, Finland (group O5, Fig. 1). We also sampled contemporary Danish River Vida whitefish, which are stocked into the German River Treene (draining into the North Sea) [35] (referred to as *C.* cf. *maraena*) (group O6, Fig. 1). To test the “devil hypothesis”, we added samples of all four Lake Constance whitefish populations (i.e. *C. wartmanni*, *C. arenicolus*, and *C. macrophthalmus* from Upper and Lower Lake Constance, group O7, Fig. 1, Table 1, Additional file 1: Table S1). After having resolved the genetic structure among these 15 contemporary populations, we compared the molecular markers of historical specimens (obtained from museums) of *C. holsatus* from Lakes Schaal and Selenter (*N* = 4, group O8, Fig. 1), and of historical *C. oxyrinchus* from the Rivers Rhine and Schelde (*N* = 14, group O9, Fig. 1, Additional file 1: Table S1) with the genetic signatures of the contemporary populations.

**Table 1:** Overview on *Coregonus* populations analysed

Population origin	Species	N	A_N_	A_R_	F_IS_	P Het
Contemporary populations						
O1_Lake Selenter	*C. maraenoides*	30	8.8	5.4	0.099	< 0.0001
O1_Lake Keller	*C. maraenoides*	24	7.6	4.8	0.188	< 0.0001
O1_Lake Poenitzer	*C. maraenoides*	32	7.8	5.0	0.147	< 0.0001
O1_Lake Schaal	*C. maraenoides*	50	9.8	5.2	0.159	< 0.0001
O2_Lake Peipsi	*C. maraenoides*	18	7.0	4.9	0.048	0.033
O3_Lake Drewitz	*C. holsatus*	20	5.5	3.9	0.134	0.002
O4_Achterwasser	*C. maraena*	24	7.0	4.7	0.057	0.001
O4_River Schlei	*C. maraena*	20	6.1	4.2	0.041	0.025
O4_River Trave	*C. maraena*	30	7.3	4.9	−0.004	0.617
O5_Oulu (Baltic Sea)	*C. widegreni*	25	9.0	5.5	0.072	< 0.0001
O6_River Treene	*C.* cf. *maraena*	20	6.2	4.4	0.072	0.007
O7_Lake Constance_Blaufelchen	*C. wartmanni*	7	3.8	3.4	0.060	0.222
O7_Lake Constance_Sandfelchen	*C. arenicolus*	9	3.5	3.1	0.029	0.119
O7_Lake Constance_Weißfisch	*C. macrophthalmus*	16	4.2	3.3	0.165	< 0.0001
O7_Lake Constance_Gangfisch	*C. macrophthalmus*	18	5.2	3.8	0.270	< 0.0001
Historical samples						
O8_Lakes Schaal, Selenter	*C. holsatus*	4				
O9_Rivers Rhine, Schelde	*C. oxyrinchus*	14				
Total		361				

Origin O1-O7: contemporary, origin O8-O9 historical whitefish (*Coregonus*) populations. Lake or river origin, scientific name, sample size (N), mean number of alleles (A_*N,*_), mean allelic richness normalized to a sample size of *N* = 7 (A_*R*_), inbreeding coefficient (*F*_*IS*_), P-values for heterozygote deficits (P Het)

### Sampling

All fish from contemporary populations were obtained from local fishermen immediately after catch in 2004 and 2005. One pectoral fin per fish was clipped and instantaneously fixed in ethanol for genetic analyses. All samples were identified to species by JF. Voucher material of all whitefish analysed is deposited in the Zoological Museum of Berlin, Germany (ZMB). From Lake Peipsi we obtained tissue from eight fish only, but successfully extracted DNA from dried scales of another 10 individuals. Historical tissue material from *C. holsatus* originates from the ZMB: two individuals from Lake Selenter and two from Lake Schaal. Origin of historical tissue material from 14 *C. oxyrinchus* specimens caught in Rivers Rhine and Schelde is listed by Freyhof & Schöter [29].

### Microsatellite analysis

Total DNA was extracted from fin tissue using the QIAGEN® DNeasy kit following the protocol of the supplier. For historical samples, the tissue was soaked in repeatedly changed TE buffer for 48 h prior to extraction and the concentration of proteinase K and the time of tissue digestion was tripled for extraction [36]. DNA concentration was determined spectrographically and aliquots diluted to a final concentration of ~ 20 ng μl^− 1^ (100 ng μl^− 1^ for historical samples). All individuals were typed at 11 polymorphic microsatellites following the protocol developed by Bittner et al. [15], supplemented by two further microsatellite loci. The two multiplex sets, i.e. simultaneous PCR amplification of multiple markers, were composed of the following loci: set 1 consisted of C2–157 [37], CoCl6, CoCl10, CoCl49, CoCl61, CoCl68 [38], and BWF1 [39]; set 2 contained CoCl4, CoCl18, CoCl45, CoCl224 [38], BWF2 [40], and CoCl-Lav52 [38]. The forward primers were labelled with fluorescent dyes (Applied Biosystems Inc., ABI): C2–157 and CoCl18 with PET™, CoCl6, CoCl10 and CoCl4 with NED™, BWF1, CoCl68, BWF2 and CoCl45 with VIC™, CoCl49, CoCl61, CoCl224, and CoCl-Lav52 with 6-FAM™. The Qiagen Multiplex PCR kit was used for PCR amplification according to the recommendations of the manufacturer.

PCR amplification was carried out in 10 μl reaction volumes, containing 5 μl QIAGEN® Multiplex PCR mastermix, 3 μl H_2_O (PCR grade), 1 μl DNA (~ 20 ng μl^− 1^) and 1 μl Primermix (~ 2 pmol μl^− 1^ each primer). The thermocycling profile started with an initial denaturation step at 95 °C for 15 min, followed by 30 cycles of 30 s at 94 °C, 90 s at 57 °C, 90 s at 72 °C and ended with a final extension step of 10 min at 72 °C. A quantity of 1.5 μl of a 1:2 dilution of the PCR reaction were added to a mix of 19 μl Hi-Di Formamide (ABI) and 1 μl LIZ™ size standard (ABI), and denatured fragments were resolved on an automated DNA sequencer (ABI 3100). Genotypes were determined with the software Gene Mapper™ version 4.0 (Applied Biosystems Inc). As a quality control for PCR and fragment analyses, DNA of two already previously genotyped individuals was amplified and analysed with any batch of 94 individuals. If loci or alleles of these two control samples were missing or differed from previous results, the fragment analysis was repeated for all samples per batch. If still alleles were missing, the PCR and fragment analysis were repeated. Analyses of individuals with missing genotypes (at one or several markers) were also repeated until completion in a similar fashion. To estimate repeatability in addition, 9.7% (35 of the 361 individuals) were analysed twice at all 13 loci and 10 mismatches were found, which relates to an overall genotyping error rate of 2.2%, not accumulated on specific loci.

### Statistical data analyses

All analysis and graphical output were performed in R version 3.4.2 [41]. Genetic diversity was quantified as mean number of alleles A_N_ and allelic richness A_R_, i.e. the allele number corrected for the smallest sample size among contemporary populations (*N* = 7) [42], by PopGenReport 3.0 [43]. Allele frequency-based correlations (inbreeding coefficient F_IS_) and population-wide deviations from Hardy–Weinberg equilibrium (HWE) were tested by the exact (probability) test using genepop_in_R based on Genepop 4.7.0 [44, 45]. The probabilities for locus-specific deviations from HWE [46] per population were corrected by the false discovery rate for multiple tests [47], completed by U-tests on excess of homozygotes. Finally, a global test for linkage disequilibrium between loci as based on the index of association with an approximation for the number of loci rD [48] was run in poppr 2.81.1 [49, 50].

To infer population structure by determining the number of clusters observed without prior knowledge, we applied Discriminant Analysis of Principal Components (DAPC) [51, 52], a model-free approach, which therefore does not require panmictic populations and unlinked markers [52]. DAPC aims to provide an efficient description of genetic clusters using a few synthetic variables. These are constructed as linear combinations of the original variables (alleles) which have the largest between-group variance and the smallest within-group variance. To define the number of principal components (PCs) retained in the analysis, we applied cross-validation which provides an objective optimization procedure for identifying the trade-off between retaining too few and too many PCs in the model. These calculations were run in adegenet 2.1.0 [53, 54]. From the DAPC, individual membership probabilities for fish from the 15 contemporary populations could be calculated as based on the retained discriminant functions, which are the linear combinations of original variables. To contrast within- and between-population variance for the 15 contemporary populations, we ran Analysis of Molecular Variance (AMOVA) [55] as implemented in the R-package ‘pegas’ [56].

To account for potential bias in individual assignment to DAPC-clusters from locus-specific deviations from HWE, we re-run the DAPC without the four loci showing the most frequent deviations from HWE (see results). Subsequently, we estimated the probability of assignment to the population of origin for each individual, and calculated the mean self-assignment for each population. The mean self-assignment as based on all 13 loci was compared with the mean self-assignment as based on the 9 loci excluding those with substantial deviations from HWE by Pearson’s correlation.

Population differentiation was estimated by F-statistics [57] between populations (pairwise θ). Significance of differentiation was assessed through the calculation of 95% confidence intervals (CI) using a bias-corrected bootstrapping method with 1000 permutations in DiveRsity 1.9.90 [58], with CIs including zero considered non-significant.

Furthermore, using the allele loadings, the individuals from historical populations (which have been excluded from the DAPC analysis) could be plotted onto the factorial DAPC plane, and we could derive their membership probabilities to the defined clusters. To support the DAPC analyses on historical individuals, we ran an alternative likelihood-based assignment procedure in Geneclass2 [59], as based on the Bayesian criterion by Baudouin & Lebrun [60].

Finally, to detect genetic relationships among all 15 contemporary and two historical populations, Nei’s genetic distances (D_A_) [61] were depicted as an unrooted neighbor-joining (NJ) dendrogram using poppr 2.6.1. [49]. To infer the effect of locus-specific deviations from HWE, another NJ dendrogram was calculated as based on the reduced number of 9 loci.

## Results

### Genetic structure within populations

Sample sizes were different between populations (range 7–50 within contemporary populations, Table 1). All microsatellites were variable in all populations, with 7–33 alleles per locus across all populations. Allelic richness A_R_, the mean number of alleles per locus and population adjusted to the minimum *N* = 7, varied little among populations (Table 1). The lowest mean values (3.1–3.8) were found in Lake Constance whitefish which had the smallest sample sizes, and the highest value (5.5) was found in *C. widegreni* from the Baltic Sea coast (Oulu). In all but one sample, the expected heterozygosity across all loci was higher than the observed heterozygosity (i.e. F_is_ > 0, see Table 1). U-tests revealed significant heterozygote deficiencies in all populations except for *C. wartmanni* and *C. arenicolus* from Lake Constance and *C. maraena* from River Trave.

The number of deviations from HWE tested individually for all 13 analyzed microsatellite loci and corrected by the false discovery rate for multiple tests varied between populations (Additional file 1: Table S2). The *C. maraenoides* population from Lake Schaal and the *C. maraena* populations of Rivers Schlei and Trave had deviations from HWE in four microsatellites, whereas *C. widegreni* (Oulu) and *C. c.f. maraena* from River Treene did not show any deviation from HWE (Additional file 1: Table S2). Most deviations from HWE occurred in the markers BWF1 (*N* = 7), BWF2 (*N* = 6), C2–157 (*N* = 4) and CoCl224 (*N* = 3) (Additional file 1: Table S2). Therefore, we repeated several subsequent tests by excluding these four loci (see below). The global test for linkage disequilibrium indicated that the alleles were weakly linked across the 13 loci (rD = 0.023, *P* = 0.001 based on 999 permutations). The strongest associations were found between the loci CoCl18 and CoCl4 (rD = 0.092), BWF1 and CoCl61 (rD = 0.086) and between CoCl4 and CoCl61 (rD = 0.082).

### Genetic structure between populations

**Fig. 2:**
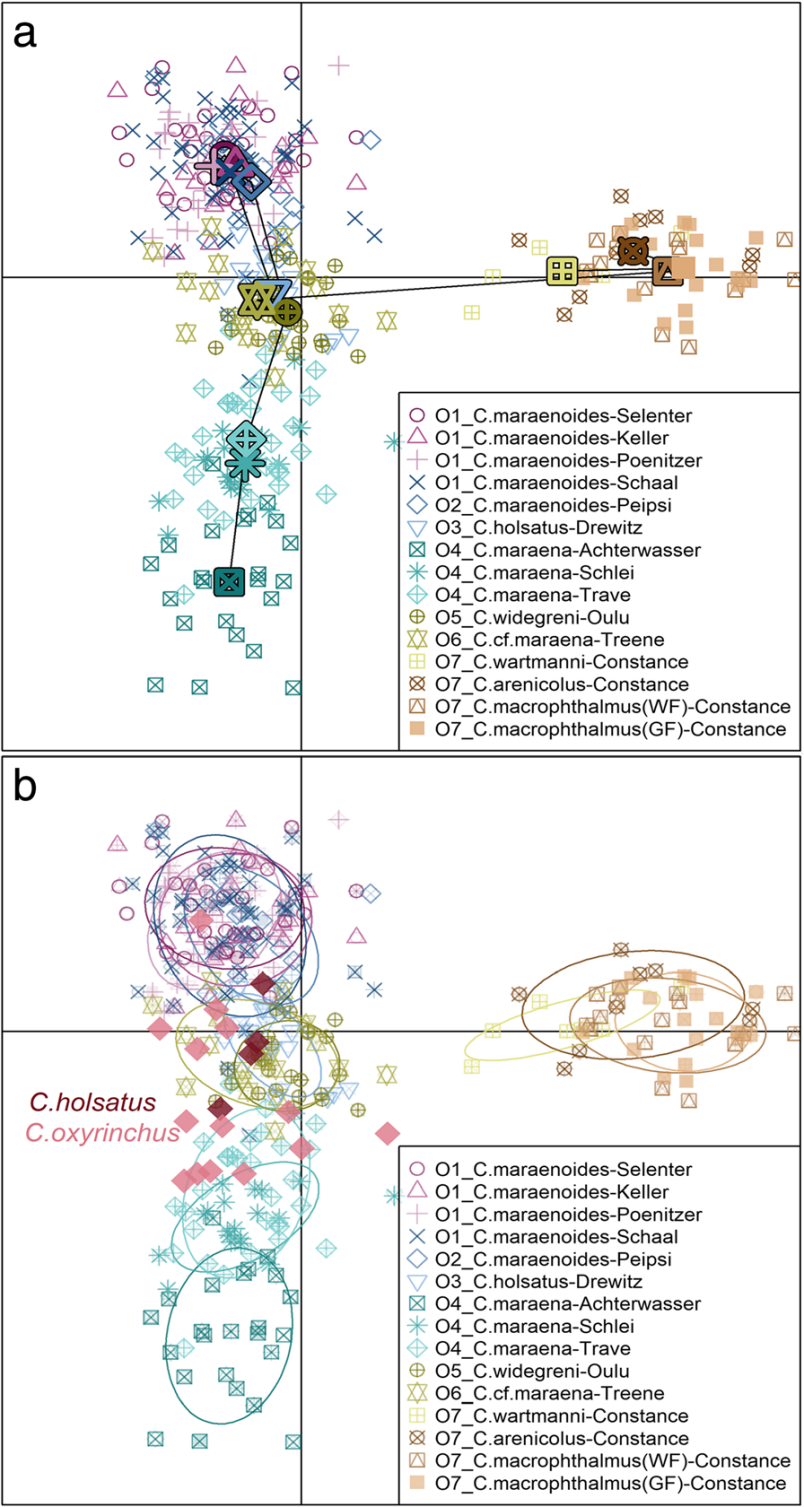
**a** Axes 1 and 2 of Discriminant Analysis of Principal Components (DAPC) of the 343 individuals of whitefishes from 15 contemporary populations. The center of each group is indicated by large symbols, the minimum spanning tree based on the (squared) distances between populations within the entire space is indicated by lines. **b** The same as in A, but populations reflected as inertia ellipses, and the 18 historical individuals from two populations (*C. holsatus* from Lakes Schaal and Selenter, *N* = 4, and *C. oxyrinchus* from Rivers Rhine and Schelde, *N* = 14) obtained from museums plotted onto the DAPC plane. For population names and geographical origin (O1-O9), see Table 1

Discriminant Analysis of Principal Components (DAPC) was run with the cross-validated optimum number of 80 principal components (cumulated variance 93.2%) and six discriminant functions and differentiated the 343 individuals from the 15 contemporary populations along the first two discriminant axes (Fig. 2a). All four Lake Constance whitefish populations were separated from the other 11 populations along axis 1. Along axis 2, *C. maraena* from Achterwasser and Rivers Trave and Schlei were opposed to the northern whitefish populations from the German Lakes Schaal, Poenitzer, Keller and Selenter and the Russian/Estonian Lake Peipsi. Lake Drewitz whitefish, *C. widegreni* (Oulu) and *C.* cf. *maraena* from River Treene were placed in between *C. maraena* and northern lake whitefish populations (Fig. 2a). Pairwise θ was significantly larger than zero for all population pairs except between some populations of the north-German lakes (Lakes Schaal, Poenitzer, Keller, Selenter, and Russian/Estonian Lake Peipsi) and among Lake Constance *C. macrophthalmus* and the other three Lake Constance populations, as indicated by 95% CI of θ, which included zero for these pairs (Additional file 1: Table S3). AMOVA indicated that the among-populations variance (10.3%, d.f. = 14, *P* < 0.0001) was greater than the among-individuals within-populations variance (9.1%, d.f. = 328, P < 0.0001).

**Fig. 3:**
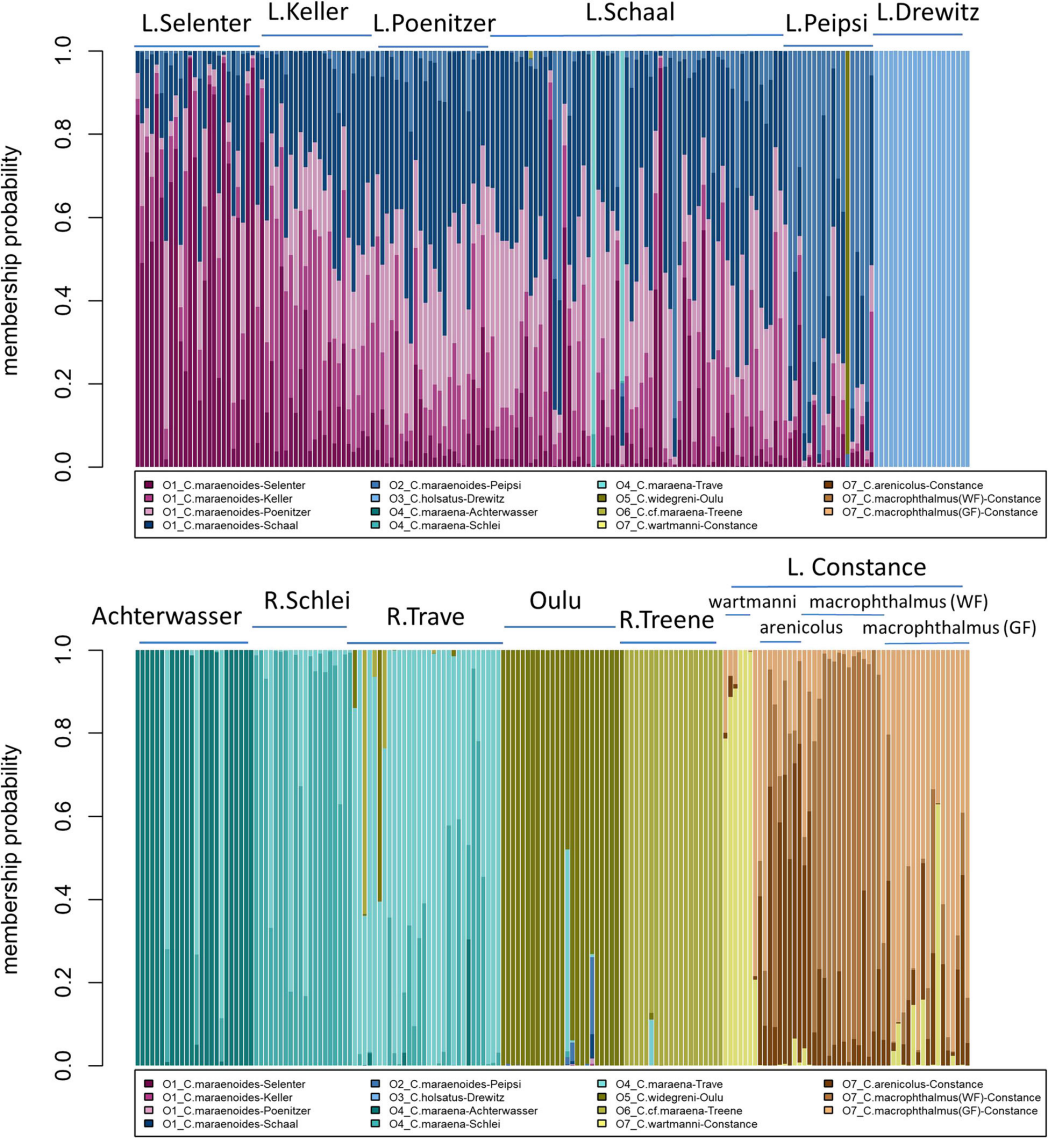
Individual membership probabilities as obtained from the Discriminant Analysis of Principal Components of 343 individuals from the 15 contemporary populations of whitefish. Columns represent individuals and the different colours within each column represent the probability of membership of that individual to a specific population, as indicated in the legend. Sampling locations of the individuals are indicated above the bars

Individual membership probabilities, as calculated from the DAPC, indicated strong admixture between the four north German lake whitefish populations (Lakes Selenter, Keller, Poenitzer, Schaal) and Lake Peipsi (Fig. 3). Mean individual self-assignment to the population of origin in this group of lakes ranged between 28% (Lake Keller) and 54% (Lake Selenter). In contrast, all whitefish from Lake Drewitz, Oulu and River Treene had mean individual self-assignment rates > 95%. Some admixture was also documented between the four populations from Lake Constance (mean self-assignment rates between 50 and 82%) (Fig. 3). There were a few single individuals with strongly deviating membership probabilities relative to their population of origin (Fig. 3). For example, two individuals of the *C. maraenoides* population of Lake Schaal were assigned to *C. maraena* from River Trave, and one individual from the *C. maraenoides* Lake Peipsi population was primarily assigned to *C. widegreni* from Oulu. Furthermore, potential admixture between *C. maraena* from River Trave, *C. widegreni* from Oulu and *C.* cf. *maraena* from River Treene was suggested for a few individuals (Fig. 3).

**Fig. 4:**
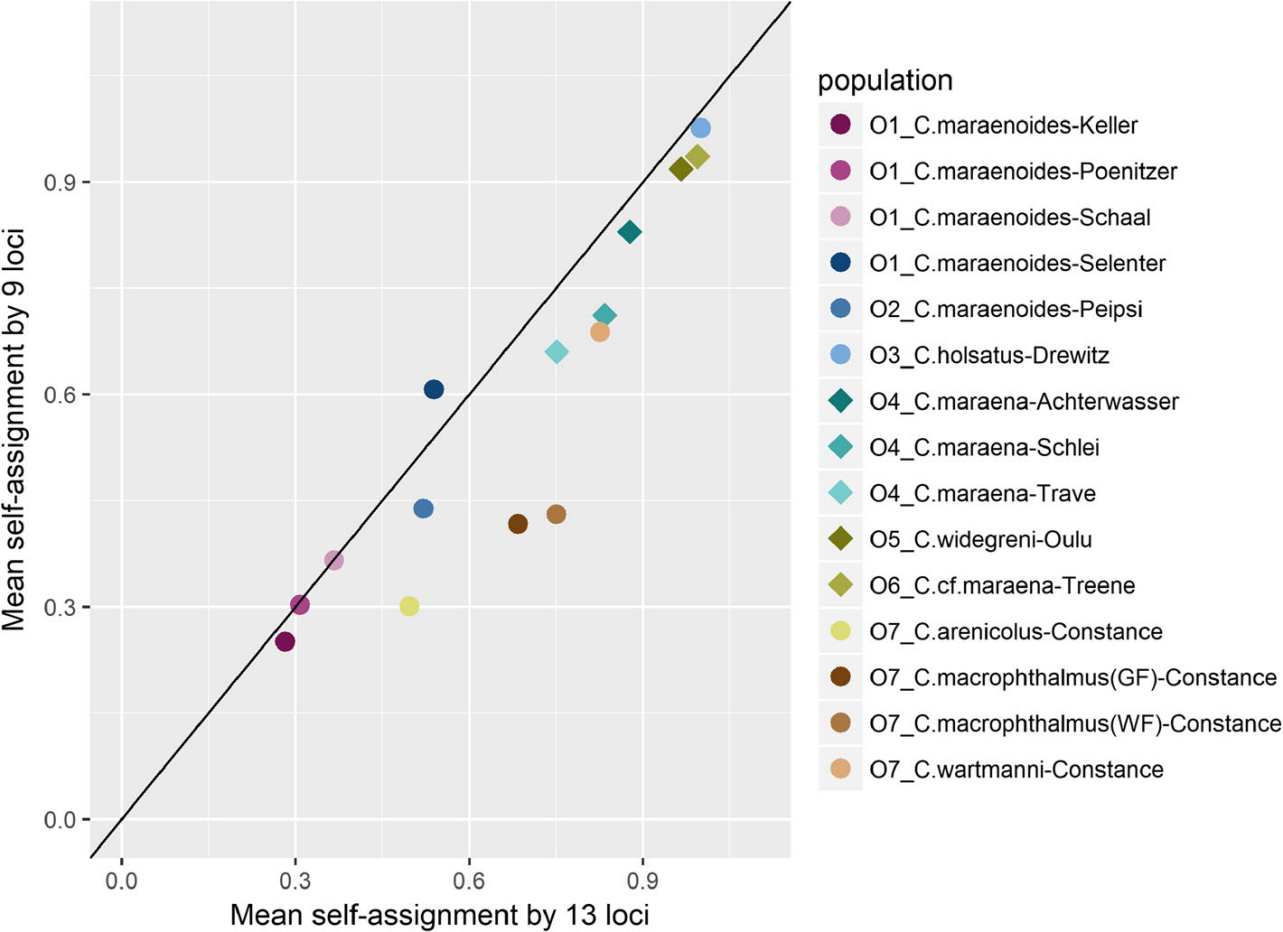
Mean individual self-assignment to the population of origin as obtained from the Discriminant Analysis of Principal Components of 343 individuals from the 15 contemporary populations of whitefish, based on either all 13 microsatellites (x-axis) or by excluding the four microsatellites with strong deviations from Hardy-Weinberg equilibrium (y-axis). Lake-resident populations are indicated by circles, whereas anadromous populations are indicated by diamonds. Data points are slightly jittered vertically to avoid overplotting. The black line represents the line of identity (1:1)

To verify that deviations from HWE did not contribute substantially to the individual assignments, we repeated the DAPC by excluding the four loci with most frequent HWE deviations. There was a strong correlation between the mean individual self-assignment rates as based on the DAPC of 9 loci and the self-assignment based on all 13 loci (Pearson’s *r* = 0.92, *P* < 0.0001). For most of the populations, the mean self-assignment rate slightly declined by excluding the four loci, but remained > 90% for whitefish from Lake Drewitz and River Treene (Fig. 4).

**Fig. 5:**
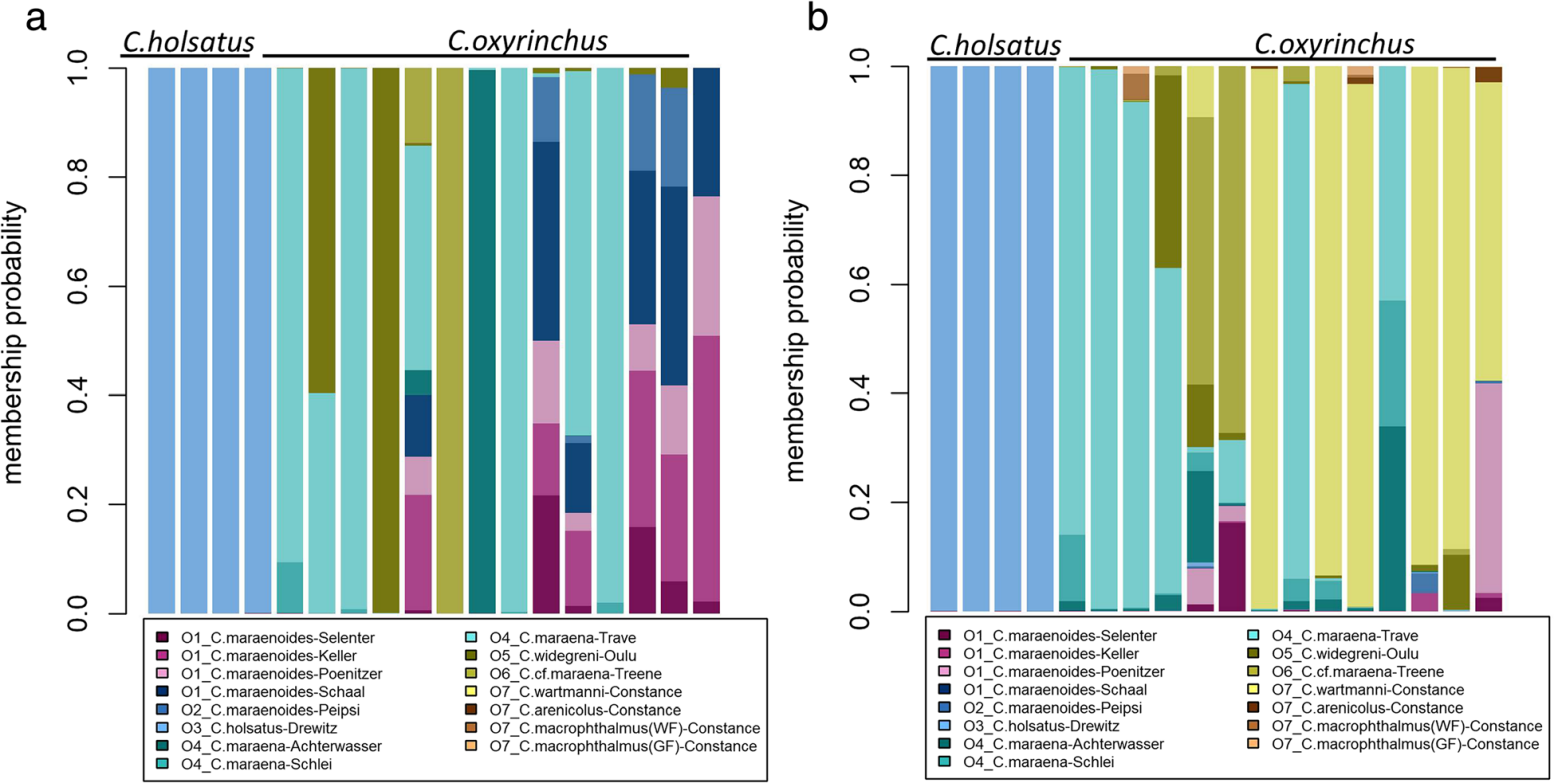
Individual membership probabilities as obtained from the Discriminant Analysis of Principal Components (**a**) or ranked likelihoods by Bayesian assignment in Geneclass2 (**b**) of 18 individuals from the historical populations of whitefish (*N* = 4 lake-resident *C.holsatus*, *N* = 14 anadromous *C. oxyrinchus*). Columns represent individuals and the different colours within each column represent the probability of membership of that individual to a specific contemporary population, as indicated in the legend

When the 18 historical individuals were plotted onto the DAPC plane, the four *C. holsatus* samples from Lakes Schaal and Selenter were located close to the contemporary Lake Drewitz and northern lake whitefish populations, whereas the 14 historical *C. oxyrhinchus* samples were scattered more broadly along axis 2 (Fig. 2b). The four historical *C. holsatus* individuals were all assigned by 100% to the contemporary Lake Drewitz population (Fig. 5a). In contrast, only seven of the 14 historical *C. oxyrinchus* individuals were assigned with > 90% probability to one of the contemporary populations (*N* = 5 to *C. maraena* from River Trave (4) or Achterwasser (1), *N* = 1 to *C. c.f. maraena* from River Treene, *N* = 1 to *C. widegreni* from Oulu) (Fig. 5a). For the remaining seven individuals, maximum assignment to any contemporary lake or anadromous population did not exceed 67% (Fig. 5a). An alternative likelihood assignment of the historical samples as based on Geneclass2 confirmed the 100% correspondence between historical *C. holsatus* and contemporary Lake Drewitz whitefish (Fig. 5b). By Geneclass2, six out of 14 historical *C. oxyrinchus* were dominantly assigned to River Trave, and two out of 14 to River Treene (Fig. 5b). The remaining six historical *C. oxyrinchus* were dominantly assigned to Lake Constance *C. wartmanni* (Fig. 5b), in contrast to the assignment by DAPC, which found higher probabilities of assignment to north-German lake populations (i.e., to *C. maraenoides*, Fig. 5a).

**Fig. 6:**
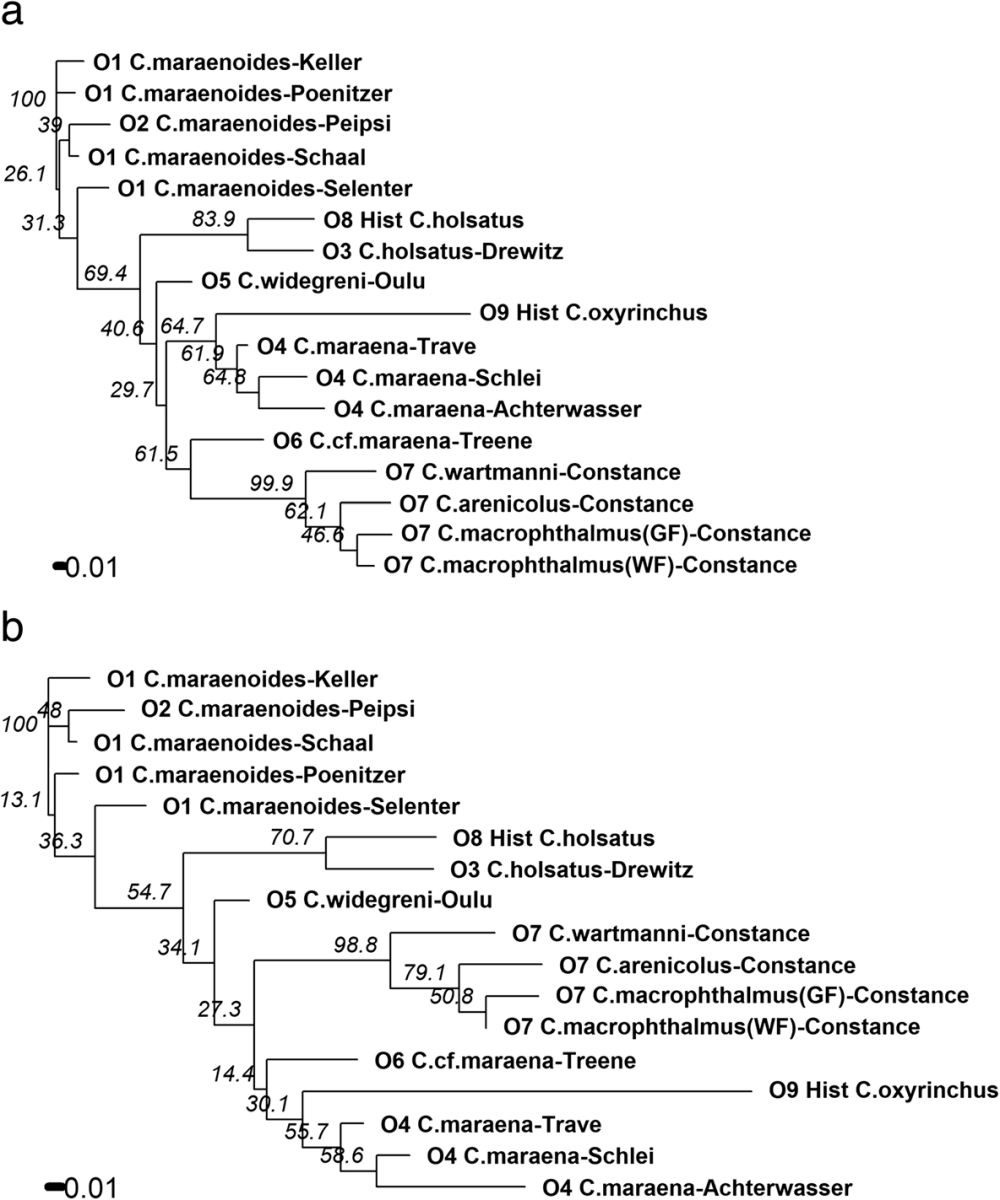
Unrooted neighbor-joining phylogram of the 15 contemporary and two historical (Hist) whitefish populations based on Nei’s standard genetic distance, calculated for 13 microsatellites (**a**), and the same phylogram calculated by excluding the four loci with strong deviations from Hardy-Weinberg equilibrium (**b**). Numbers represent bootstrap values based on 1000 repetitions. For population names and groups of geographical origin (O1-O9) see Table 1

Similarities of the 15 contemporary and two historical populations were finally depicted as an unrooted neighbor-joining phylogram of Nei’s genetic distance (Fig. 6). Lakes Schaal and Peipsi were grouped together, with low support (bootstrap value 39%) of branching (Fig. 6a). In contrast, Lake Selenter was grouped closer to Lake Drewitz, but with low support (31.3%). Historical and contemporary *C. holsatus* (Lake Drewitz) were only weakly segregated from the north German Lakes Keller, Schaal, Poenitzer, Selenter and Russian/Estonian Lake Peipsi (31.3%), but more strongly segregated (69.4%) from Lake Constance whitefish and from the anadromous whitefish populations (Fig. 6a). The historical *C. oxyrinchus* grouped closest with *C. maraena* from Achterwasser and Rivers Schlei and Trave with moderate bootstrap support (64.7%). River Treene *C.*cf. *maraena* grouped with Lake Constance whitefish populations (61.5%). However, the overall segregation between all anadromous populations and Lake Constance whitefish was weak (29.7–40.6%, Fig. 6a). If the four loci deviating most frequently from HWE were removed, the NJ-tree remained essentially identical (Fig. 6b). However, River Treene *C.*cf. *maraena* grouped closer to historical *C. oxyrinchus* and *C. maraena* from Achterwasser and Rivers Schlei and Trave. Nevertheless, the overall segregation between all anadromous populations and Lake Constance whitefish remained weak (range of bootstrap supports 14.4–34.1%, Fig. 6b).

## Discussion

Our results demonstrate that the contemporary whitefish population of Lake Schaal is non-native, and does no longer correspond to the originally described *C. holsatus* from this lake. The most likely origin of the fish currently occurring in Lake Schaal is Lake Peipsi (= *C. maraenoides*), from which whitefish was stocked into Lake Schaal. A historical translocation of whitefish from Lake Constance into Lake Schaal (the “devil hypothesis”) could not be confirmed, because the molecular markers of both contemporary (*C. maraenoides*) and historical (*C. holsatus*) Lake Schaal whitefish were distinct from those of all four contemporary Lake Constance populations. Furthermore, there is no genetic evidence that anadromous whitefish populations of the Baltic and North Sea basins (= *C. maraena*) contributed to stocking in Lake Schaal. These studied anadromous whitefish are genetically closely related, but none of them was genetically truly identical with *C. oxyrinchus* historically occurring in the Rivers Rhine and Schelde, supporting the view of Freyhof & Schöter [29] that *C. oxyrinchus* is extinct. Accordingly, our application of genetic markers to a mixture of contemporary and historical samples may guide future conservation practice to protect evolutionarily significant whitefish populations.

The population structure found by all applied statistical methods was fairly similar. Both the dendrogram as based on Nei’s genetic distances between populations and the individual-based DAPC showed strong allelic differences in particular between Lake Constance whitefishes, the whitefishes from north German lakes (= *C. maraenoides*), and *C. maraena*. Accordingly, it is unlikely that the limitations of the dataset with in part small sample sizes and strong deviations from HWE have biased the results. This conclusion is supported by the additional analyses, which excluded the four loci most frequently deviating from HWE. Both the mean proportion of self-assignment in the DAPC, reflecting the individual level, and the NJ tree of genetic distances, reflecting the population level, remained almost unchanged when the analyses were repeated with nine instead of 13 loci.

The reasons for the substantial heterozygote deficits at some loci and the weak, but significant global linkage disequilibrium are not clear. The quality control of the genotyping procedures did not reveal any technical abnormalities. Strong deviations from HWE were found in particular in whitefish populations that have been stocked repeatedly (Lake Schaal, Lake Constance, Rivers Schlei and Trave). Heterozygote deficits may be caused by population substructuring (Wahlund effect) [62, 63], and Wahlund effects have been documented repeatedly in lake populations of the Salmoniformes order [64–66]. Stocking may contribute to population substructures if natural reproduction in the system is maintained and stocked and naturally reproduced individuals do not form panmictic populations because of local adaptation and polymorphism. It is possible that potential population substructures were not adequately covered by our samples, because genotyped individuals were taken from the commercial fisheries catches, and not obtained by 3D stratified sampling as recommended for polymorphic populations [67].

We found evidence for the existence of six genetically distinct groups of whitefish populations. The first three groups consisted of the four Lake Constance populations, the three *C. maraena* populations and the five combined north German lake and Lake Peipsi populations (= *C. maraenoides*). These three groups separated clearly on the DAPC plane. In addition, whitefish from Lake Drewitz (= *C. holsatus*), Oulu (= *C. widegreni*) and River Treene (= *C.* cf. *maraena*) each were depicted as different populations by significant θ in pairwise comparisons and the highest proportions of individual self-assignment, although these populations grouped closely together on the DAPC plane. Most importantly in the context of this study, all Lake Constance whitefish were clearly distinguished from the contemporary Lake Drewitz whitefish and the historical *C. holsatus* from Lakes Schaal and Selenter. This unambiguously falsifies the assumption of Winkler et al. [68] that historical *C. holsatus* from Lakes Schaal and Selenter originate from stocking of whitefish from Lake Constance. Therefore, we have to reject the “devil hypothesis” finding no support that *C. holsatus* was based on introduced Lake Constance whitefish.

For conservation purposes, *C. holsatus* was accordingly preliminarily treated as a unique and endemic conservation unit classified as “extinct in the wild” [33] as it did not survive within its native historical range. The extinction in Lake Schaal was confirmed by our molecular data. However, the original *C. holsatus* has survived by translocation. Due to the clear morphological and genetic similarities of contemporary Lake Drewitz whitefish with the four historical *C. holsatus* specimens we classify contemporary Lake Drewitz whitefish as *C. holsatus* and confirm that *C. holsatus* from Lake Schaal was the initial source for the introduced and presently established whitefish in Lake Drewitz [26]. The genetical assignment of the historical *C. holsatus* from Lakes Schaal and Selenter to contemporary *C. holsatus* from Lake Drewitz was independently found by two statistical approaches (DAPC and Geneclass2), and hence is considered reliable. The whitefish in Lake Drewitz have to be classified as non-native since this species did not inhabit the lake historically. Nevertheless, Lake Drewitz whitefish have to be protected from any kind of stocking or other management endangering the genetic integrity of the species.

The contemporary Lake Schaal whitefish and the whitefish in the nearby northern German lakes (Poenitzer, Keller, and Selenter) can be identified as Lake Peipsi whitefish *C. maraenoides*, in contrast to the common designation as European lake whitefish *C. lavaretus* (e.g., [69]). *C. maraenoides* has been stocked to various lakes in north western Germany since the 1930s [26, 27], and has obviously replaced *C. holsatus* in Lake Schaal, and to a lesser extent also in Lake Selenter. The temporal and causal aspects of these replacements are unknown. It is possible that *C. maraenoides* outcompeted *C. holsatus*, or that both species hybridized to some extent, or that *C. holsatus* declined due to the eutrophication of both lakes in the twentieth century [70] before or after stocking with *C. maraenoides*. Consequently, following Winkler et al. [68], it can be recommended to exclude the contemporary Lakes Schaal (and Selenter) whitefish from national conservation efforts due to their presumed non-native origin.

The fact that the four historical individuals of *C. holsatus* from Lakes Schaal and Selenter were projected close to the whitefish from Lake Drewitz but also close to the contemporary north German lake populations (i.e., *C. maraenoides*) onto the DAPC plane may point to the presence of some remnant hybridized genetic material in the contemporary Lakes Schaal and Selenter populations. Admixture with stocked material was also suggested by the membership probabilities as calculated from the DAPC, and by the weak bootstrap support of branching between *C. maraenoides* and *C. holsatus*. Similarly, supplementary stocking in a south-eastern Finnish watercourse has contributed to a high degree of genetic admixture between originally distinct whitefish [71], and translocation of Baltic whitefish into Austrian Alpine lakes has created hybrids [72]. In contrast, weak introgression from Siberian *Coregonus peled* into local lake whitefish populations was found in Poland, despite artificial fertilization in hatcheries [73]. Accordingly, the degree of hybridization between native and introduced species of whitefish seems to be difficult to predict.

In 1948, Lenz [26] reported that *C. maraenoides* is better adapted to eutrophic lakes and that stocking of artificially reared *C. maraenoides* gained higher yields than stocking of *C. holsatus*. Thus it is possible that *C. maraenoides* was preferred for artificial reproduction by commercial fishermen, since both species are easy to distinguish by mouth and body shape. When both lakes became strongly eutrophic preventing ultimately the natural reproduction of all whitefish species [74], the native *C. holsatus* may have vanished while the introduced *C. maraenoides* was kept by artificial reproduction. Stocking measures in combination with anthropogenically induced environmental changes have been presumed responsible for the extinction of several other indigenous populations of Salmoniformes in Europe [19, 34, 70, 75, 76]. In Lake Drewitz, *C. holsatus* may have proliferated because in this lake the only other coregonid species occurring is the much smaller vendace *C. albula* [19], whereas whitefish were absent, and *C. maraenoides* was never stocked.

To test Thienemann’s assumption that *C. holsatus* is an independent species we compared the historical *C. holsatus* specimens with contemporary whitefish species in North and Central Europe. Our limited dataset does not allow resolving the diversity of all northern whitefish, but we can unambiguously refute that *C. holsatus* is the same evolutionary unit as any other contemporary whitefish studied here. We could not confirm that *C. holsatus* and *C. widegreni* are conspecific [22]. The Finnish sea-spawning whitefish from Oulu named as *C. widegreni* included into this study was genetically significantly differentiated from the contemporary Lake Drewitz whitefish (*C. holsatus* translocated from Lake Schaal). Furthermore, in the NJ tree, the Oulu sea-spawning whitefish grouped with contemporary anadromous whitefish from the Baltic (Achterwasser, Schlei and Trave), the North Sea (River Treene) and the Lake Constance whitefish populations. More studies are needed to identify the phylogeography of *C. widegreni* in the context of other Scandinavian and Russian anadromous whitefish species.

As a consequence of the broad variety of whitefish populations studied, we could also explore the genetic relationships of anadromous whitefish from the south-western Baltic and eastern North Sea basins for conservation purposes. In the south-western Baltic Sea basin, only one population of anadromous whitefish has survived in the lower Peene/Odra drainage [77]. This species was preliminarily identified as *C. maraena* [29]. The whitefish from Achterwasser included in our study belong to this population. Since 1992, fish from this population have been stocked into the German Rivers Schlei and Trave [27], and the admixture between these three populations is reflected in the individual assignment as based on the DAPC. In contrast, in the entire North Sea basin, anadromous whitefish have only survived in the Danish river Vida [78, 79]. Since 1987, brood-stock of this Vida population has been used for several reintroduction programs in Danish and German North Sea tributaries including the River Rhine [80]. River Vida is also the origin of the whitefish from River Treene included in our study [35, 80]. In the DAPC plot, the River Treene whitefish were closely located to *C. widegreni* from Oulu and *C. holsatus* from Lake Drewitz and next to River Trave *C. maraena*, but the proportion of individual self-assignment to River Treene in the DAPC was almost 100%. In the NJ tree, River Treene whitefish primarily grouped with Lake Constance whitefish populations, whereas the next branch consisted of the anadromous whitefish *C. maraena* from the German Baltic Sea coast (Achterwasser, Schlei and Trave). Previously, the River Treene whitefish have likewise preliminarily been identified as *C. maraena* [29], and therefore it was suggested that all contemporary anadromous whitefish populations in the Baltic and North Sea can be considered as *C. maraena.* This conclusion was supported by Hansen et al. [30, 81] who found only small differences of mitochondrial DNA and microsatellite markers between whitefish from the Baltic and North Sea basins.

A recent admixture analysis suggested a hybrid zone between River Vida whitefish, *C. maraena* from River Peene/Odra (identical to Achterwasser and Rivers Schlei and Trave in our study) and whitefish from north German lakes, including Lake Poenitzer, potentially caused by erroneous stocking with non-native genotypes [69]. Admixture may also be caused by escaped larvae of River Treene whitefish, which were raised in netcages in Lake Keller [80]. The assignment of fish as based on the DAPC in our study supported that single individuals may represent hybrids between whitefish in Lake Schaal (identified as *C. maraenoides* in our study) and *C. maraena* from River Trave. However, by sequencing the whole mitochondrial genome, recent strong reproductive isolation between Danish North Sea populations from River Vida and the Baltic lake whitefish *C. lavaretus* complex (including fish from Achterwasser, named as *C. maraena* in our study) has been demonstrated [82]. This study [82] corresponds with the distinct branching in the NJ tree between the contemporary *C. c.f. maraena* from the River Vida and *C. maraena* from the three Baltic Sea populations (Achterwasser, Schlei and Trave), although the bootstrap support was low (about 30%). Overall, the genetic similarity was higher between River Treene whitefish and *C. maraena* from River Trave, than between River Treene whitefish and *C. maraena* from Achterwasser, the original source of stocked whitefish in River Trave.

The 14 historical *C. oxyrinchus* individuals were assigned primarily to the contemporary *C. maraena* populations (7 of 14 individuals with > 50% assignment), whereas only one individual was assigned to the contemporary River Treene population, which is usually identified as *C. oxyrinchus*. Similarly, the historical *C. oxyrinchus* individuals were grouped next to the three *C. maraena* populations in the NJ tree. This suggests a weaker genetic differentiation between contemporary *C. maraena* and historical *C. oxyrinchus* than between contemporary anadromous River Vida/Treene and historical *C. oxyrinchus* populations. This has clear implications for the current stocking programs in the North Sea tributaries. According to our data, the fish currently stocked into the River Rhine [80] from River Vida cannot be treated as re-introduction of *C. oxyrinchus* [29], as the historical specimens of *C. oxyrinchus* are genetically fairly distinct from contemporary River Treene/Vida whitefish. However, it has to be stressed that the bootstrap support for the major branching in the NJ tree among all anadromous populations including the historical *C. oxyrinchus* was very low.

Based on morphological data, Freyhof & Schöter [29] have supported Redeke’s [83] and Thienemann’s [84] assumption that historically two species of anadromous whitefish may have been present in the North Sea basin: *C. oxyrinchus* in England and in the Rivers Rhine, Meuse and Schelde, and *C. maraena* migrating along the rivers east of the Rhine (eastern North Sea and Baltic basins). The scattered assignment of the 14 historical western houting individuals (named as *C. oxyrinchus*) to contemporary anadromous and lake-resident whitefish suggests that this distinction between *C. maraena* and *C. oxyrinchus* within the North Sea basins is less clear than assumed. We can only speculate about the background of these findings. For the nineteenth century when the historical material of *C. oxyrinchus* had been sampled, there is no reported stocking in the geographical range where *C. oxyrinchus* has occurred. However, we cannot exclude that *C. oxyrinchus* has experienced introgressive hybridisation involving genes from Lake Constance whitefish (as suggested by the assignment by Geneclass2), which have always also occurred in the lower Rhine, as well as from *C. maraena* straying from the east (as suggested by the assignment from both DAPC and Geneclass2). We encourage future in-depth studies to test for introgression of adjacent species into *C. oxyrinchus*. A broader spatial scale and exactly defined geographical origin of historical and contemporary samples from anadromous whitefishes of the Baltic and North Sea tributaries would help resolving the complex phylogeography of these northern and middle European whitefish species and populations.

## Conclusions

Stocking and translocations which have begun at least 100 years ago have modified the recent genetic signature of many whitefish species and populations. In some cases, local populations vanished and were replaced by stocked whitefish species originating from other sources. The often poorly documented stocking history and the unpredictability of stocking success contribute to the unresolved conservation status for many whitefish species and populations. To facilitate informed decisions on the conservation of evolutionarily significant units within the whitefish complex, further comparative genetic studies of contemporary and historical material are urgently needed.

## Additional file

Additional file 1: **Table S1.** Geographical coordinates (degrees), catchment, basin and mean depth and surface area (for lakes only) of aquatic systems, from which the *Coregonus* populations were sampled. **Table S2.** *P*-values of deviations from Hardy-Weinberg equilibrium per locus and population, as controlled by the false discovery rate for multiple tests. **Table S3.** Matrix of pairwise θ between the 15 contemporary whitefish populations (below diagonal), and their lower 95% confidence intervals (above diagonal). Strong structure between populations is indicated in bold, as evidenced by the lower CI not including zero. For population origin, see Table 1. L. Const = Lake Constance. (DOCX 29 kb)

## Abbreviations

AMOVA: Analysis of Molecular Variance
A_N_: Number of alleles
A_R_: Allelic richness
CI: Confidence interval
D_A_: Nei’s genetic distance
DAPC: Discriminant Analysis of Principal Components
F_IS_: Inbreeding coefficient
HWE: Hardy–Weinberg equilibrium
NJ dendrogram: Neighbor-joining dendrogram
rD: Index of association with an approximation for the number of loci
ZMB: Zoological Museum of Berlin, Germany
