# Supplementary material for: Testing the devil’s impact on southern Baltic and North Sea basins whitefish (Coregonus spp.) diversity
Source: BMC Evol Biol. 2018 Dec 29;18:208. doi: 10.1186/s12862-018-1339-2 (PMC6311081; doi:10.1186/s12862-018-1339-2)
Supplement: Supplementary file 1 — Table S1. Geographical coordinates (degrees), catchment, basin and mean depth and surface area (for lakes only) of aquatic systems, from which the Coregonus populations were sampled. Table S2. P-values of deviations from Hardy-Weinberg equilibrium per locus and population, as controlled by the false discovery rate for multiple tests. Table S3. Matrix of pairwise θ between the 15 contemporary whitefish populations (below diagonal), and their lower 95% confidence intervals (above diagonal). Strong structure between populations is indicated in bold, as evidenced by the lower CI not including zero. For population origin, see Table 1. L. Const = Lake Constance. (DOCX 29 kb) [file 12862_2018_1339_MOESM1_ESM.docx]

**Testing the devil’s impact on southern Baltic and North Sea basin whitefish**

**(*Coregonus* spp) diversity**

Thomas Mehner^1^, Kirsten Pohlmann^1^, David Bittner^2,3^ & Jörg Freyhof^1^

^1^ Leibniz-Institute of Freshwater Ecology and Inland Fisheries, Müggelseedamm 310, 12587 Berlin, Germany. E-mail: [mehner@igb-berlin.de](mailto:mehner@igb-berlin.de), [j.freyhof@igb-berlin.de](mailto:j.freyhof@igb-berlin.de), [kpohlmann@igb-berlin.de](mailto:kpohlmann@igb-berlin.de)

^2^ Department of Fish Ecology & Evolution, Centre of Ecology, Evolution and Biogeochemistry, EAWAG Swiss Federal Institute of Aquatic Science and Technology, Seestrasse 79, 6047 Kastanienbaum, Switzerland.

^3^ current address: Kanton Aargau, Departement Bau, Verkehr und Umwelt, Abteilung Wald, Jagd und Fischerei, Entfelderstrasse 22, 5001 Aarau, Switzerland

E-mail: [david.bittner@ag.ch](mailto:david.bittner@ag.ch)

**Appendix Tables**

Appendix Table 1: Geographical coordinates (degrees), catchment, basin and mean depth and surface area (for lakes only) of aquatic systems, from which the *Coregonus* populations were sampled.

| Population origin | Longitude | Latitude | Catchment | Basin | Mean depth (m) | Lake surface (km^2^) |
| --- | --- | --- | --- | --- | --- | --- |
| Contemporary populations |  |  |  |  |  |  |
| O1_Lake Selenter | 10.4476 | 54.3068 | Hagener Au | Baltic Sea | 14 | 21.0 |
| O1_Lake Keller | 10.5928 | 54.1783 | Malente | Baltic Sea | 13 | 5.6 |
| O1_Lake Poenitzer | 10.6941 | 54.0314 | Heidebek | Baltic Sea | 5 | 1.1 |
| O1_Lake Schaal | 10.9271 | 53.5855 | Elbe | North Sea | 17 | 24.0 |
| O2_Lake Peipsi | 27.4916 | 58.6766 | Narva | Baltic Sea | 7 | 3820.0 |
| O3_Lake Drewitz | 12.3588 | 53.5411 | Elbe | North Sea | 10 | 7.0 |
| O4_Achterwasser | 13.9713 | 54.0083 | Odra | Baltic Sea |  |  |
| O4_River Schlei | 9.9717 | 54.6780 | Schlei | North Sea |  |  |
| O4_River Trave | 10.8871 | 53.9607 | Trave | North Sea |  |  |
| O5_Oulu | 25.4725 | 65.0133 | Baltic Sea | Baltic Sea |  |  |
| O6_River Treene | 9.0969 | 54.3809 | Eider | North Sea |  |  |
| O7_Lake Constance_Obersee | 9.4081 | 47.6219 | Rhine | North Sea | 101 | 472.0 |
| O7_Lake Constance_Untersee | 9.0195 | 47.6894 | Rhine | North Sea | 13 | 62.0 |
| Historical samples |  |  |  |  |  |  |
| O8_Lake Selenter | 10.4476 | 54.3068 | Hagener Au | Baltic Sea | 14 | 21.0 |
| O8_Lake Schaal | 10.9271 | 53.5855 | Elbe | North Sea | 17 | 24.0 |
| O9_Rivers Rhine, Schelde | 4.2550 | 51.3430 | Rhine + Schelde | North Sea |  |  |

Appendix Table 2: P-values of deviations from Hardy-Weinberg equilibrium per locus and population, as controlled by the false discovery rate for multiple tests.

|  | O1_  L.Selenter | O1_  L.Keller | O1_  L.Poenitzer | O1_  L.Schaal | O2_  L.Peipsi | O3_  L.Drewitz | O4_Achter-wasser | O4_  R.Schlei | O4_  R.Trave | O5_Oulu | O6_  R.Treene | O7_  L.Const_BF | O7_  L.Const_SF | O7_  L.Const_WF | O7_  L.Const_GF | No. Sign.  Dev. | | |
| --- | --- | --- | --- | --- | --- | --- | --- | --- | --- | --- | --- | --- | --- | --- | --- | --- | --- | --- |
| BWF1 | 0.0006 | 1 | 1 | 0.0035 | 7.1E-09 | 0.00511 | 1 | 0.0060 | 1 | 1 | 1 | 2.06E-05 | 0.0107 | 1 | 1 |  | 7 |  |
| BWF2 | 1 | 1 | 0.0124 | 1 | 1 | 0.00093 | 1 | 0.0004 | 0.0161 | 1 | 1 | 7.09E-09 | 1 | 0.01616 | 1 |  | 6 |  |
| C2-157 | 1 | 8.9E-06 | 1 | 8.7E-06 | 0.006 | 1 | 1 | 1 | 1 | 1 | 1 | 1 | 1 | 1 | 5.04E-09 |  | 4 |  |
| CoCl10 | 1 | 1 | 1 | 0.02054 | 1 | 1 | 1 | 1 | 1 | 1 | 1 | 1 | 1 | 1 | 1 |  | 1 |  |
| CoCl18 | 1 | 1 | 1 | 1 | 1 | 1 | 1 | 1 | 1 | 1 | 1 | 1 | 1 | 1 | 1 |  | 0 |  |
| CoCl224 | 1 | 1 | 1 | 1 | 1 | 1 | 1 | 0.0003 | 0.0161 | 1 | 1 | 1 | 1 | 1 | 0.00774 |  | 3 |  |
| CoCl4 | 1 | 1 | 1 | 1 | 1 | 1 | 1 | 1 | 1 | 1 | 1 | 1 | 1 | 0.01616 | 1 |  | 1 |  |
| CoCl45 | 1 | 1 | 1 | 1 | 1 | 1 | 1 | 1 | 0.0124 | 1 | 1 | 1 | 1 | 1 | 1 |  | 1 |  |
| CoCl49 | 1 | 1 | 1 | 1 | 1 | 1 | 1 | 1 | 1 | 1 | 1 | 1 | 1 | 1 | 1 |  | 0 |  |
| CoCl-Lav52 | 1 | 1 | 1 | 0.00292 | 1 | 1 | 1 | 1 | 1 | 1 | 1 | 1 | 1 | 1 | 1 |  | 1 |  |
| CoCl6 | 1 | 1 | 1 | 1 | 1 | 1 | 0.00773 | 1 | 1 | 1 | 1 | 1 | 1 | 1 | 0.0053 |  | 2 |  |
| CoCl61 | 1 | 1 | 1 | 1 | 1 | 1 | 1 | 1 | 1 | 1 | 1 | 1 | 1 | 1 | 1 |  | 0 |  |
| CoCl68 | 1 | 1 | 1 | 1 | 1 | 1 | 1 | 2.1E-05 | 1 | 1 | 1 | 1 | 0.0025 | 1 | 1 |  | 2 |  |
|  |  |  |  |  |  |  |  |  |  |  |  |  |  |  |  |  |  |  |
| No. Sign.  Dev. | 1 | 1 | 1 | 4 | 2 | 2 | 1 | 4 | 4 | 0 | 0 | 2 | 2 | 1 | 3 |  | 28 |  |

Appendix Table 3: Matrix of pairwise θ between the 15 contemporary whitefish populations (below diagonal), and their lower 95% confidence intervals (above diagonal). Strong structure between populations is indicated in bold, as evidenced by the lower CI not including zero. For population origin, see Table 1. L.Const = Lake Constance.

| Populations | O1  L.Selenter | O1  L.Keller | O1  L.Poenitzer | O1  L.Schaal | O2  L.Peipsi | O3  L.Drewitz | O4  Achterwasser | O4  R.Schlei | O4  R.Trave | O5  Oulu | O6  R.Treene | O7  L.Const_BF | O7  L.Const_SF | O7  L.Const_WF | O7  L.Const_GF |
| --- | --- | --- | --- | --- | --- | --- | --- | --- | --- | --- | --- | --- | --- | --- | --- |
| O1_L.Selenter |  | -0.003 | -0.003 | **0.005** | **0.013** | **0.070** | **0.098** | **0.089** | **0.077** | **0.047** | **0.092** | **0.101** | **0.108** | **0.146** | **0.123** |
| O1_L.Keller | 0.015 |  | -0.010 | -0.005 | **0.002** | **0.112** | **0.087** | **0.098** | **0.064** | **0.048** | **0.070** | **0.097** | **0.115** | **0.138** | **0.128** |
| O1_L.Poenitzer | 0.011 | 0.005 |  | -0.003 | -0.004 | **0.104** | **0.093** | **0.086** | **0.067** | **0.036** | **0.078** | **0.095** | **0.115** | **0.137** | **0.130** |
| O1_L.Schaal | **0.020** | 0.007 | 0.008 |  | -0.006 | **0.110** | **0.092** | **0.096** | **0.063** | **0.035** | **0.074** | **0.102** | **0.109** | **0.138** | **0.127** |
| O2_L.Peipsi | **0.033** | **0.022** | 0.014 | 0.007 |  | **0.095** | **0.092** | **0.090** | **0.063** | **0.021** | **0.082** | **0.101** | **0.108** | **0.131** | **0.132** |
| O3_L.Drewitz | **0.095** | **0.138** | **0.129** | **0.128** | **0.124** |  | **0.139** | **0.124** | **0.145** | **0.091** | **0.149** | **0.161** | **0.188** | **0.224** | **0.196** |
| O4_Achterwasser | **0.120** | **0.106** | **0.114** | **0.111** | **0.117** | **0.166** |  | **0.030** | **0.019** | **0.073** | **0.100** | **0.110** | **0.133** | **0.153** | **0.140** |
| O4_R.Schlei | **0.116** | **0.128** | **0.112** | **0.121** | **0.123** | **0.160** | **0.058** |  | **0.012** | **0.065** | **0.113** | **0.165** | **0.156** | **0.185** | **0.181** |
| O4_R.Trave | **0.099** | **0.082** | **0.089** | **0.082** | **0.089** | **0.174** | **0.040** | **0.042** |  | **0.037** | **0.047** | **0.128** | **0.117** | **0.137** | **0.136** |
| O5_Oulu | **0.067** | **0.067** | **0.056** | **0.051** | **0.042** | **0.115** | **0.093** | **0.097** | **0.056** |  | **0.035** | **0.089** | **0.089** | **0.121** | **0.114** |
| O6_R.Treene | **0.118** | **0.095** | **0.103** | **0.093** | **0.111** | **0.183** | **0.125** | **0.156** | **0.068** | **0.056** |  | **0.102** | **0.108** | **0.119** | **0.111** |
| O7_L.Const_BF | **0.136** | **0.129** | **0.130** | **0.134** | **0.140** | **0.203** | **0.144** | **0.214** | **0.160** | **0.121** | **0.139** |  | **0.036** | **0.030** | -0.026 |
| O7_L.Const_SF | **0.143** | **0.152** | **0.149** | **0.141** | **0.153** | **0.228** | **0.171** | **0.204** | **0.158** | **0.133** | **0.153** | **0.102** |  | **0.007** | -0.003 |
| O7_L.Const_WF | **0.173** | **0.167** | **0.167** | **0.163** | **0.170** | **0.256** | **0.179** | **0.228** | **0.163** | **0.148** | **0.144** | **0.076** | **0.053** |  | -0.006 |
| O7_L.Const_GF | **0.150** | **0.152** | **0.156** | **0.151** | **0.164** | **0.230** | **0.168** | **0.222** | **0.159** | **0.138** | **0.138** | 0.023 | 0.038 | 0.022 |  |
